# Piperine Causes G1 Phase Cell Cycle Arrest and Apoptosis in Melanoma Cells through Checkpoint Kinase-1 Activation

**DOI:** 10.1371/journal.pone.0094298

**Published:** 2014-05-07

**Authors:** Neel M. Fofaria, Sung-Hoon Kim, Sanjay K. Srivastava

**Affiliations:** 1 Department of Biomedical Sciences and Cancer Biology Center, Texas Tech University Health Sciences Center, Amarillo, Texas, United States of America; 2 Cancer Preventive Material Development Research Center, College of Korean Medicine, Department of Pathology, Kyunghee University, Seoul, South Korea; Universita’ di Milano, Italy

## Abstract

In this study, we determined the cytotoxic effects of piperine, a major constituent of black and long pepper in melanoma cells. Piperine treatment inhibited the growth of SK MEL 28 and B16 F0 cells in a dose and time-dependent manner. The growth inhibitory effects of piperine were mediated by cell cycle arrest of both the cell lines in G1 phase. The G1 arrest by piperine correlated with the down-regulation of cyclin D1 and induction of p21. Furthermore, this growth arrest by piperine treatment was associated with DNA damage as indicated by phosphorylation of H2AX at Ser139, activation of ataxia telangiectasia and rad3-related protein (ATR) and checkpoint kinase 1 (Chk1). Pretreatment with AZD 7762, a Chk1 inhibitor not only abrogated the activation of Chk1 but also piperine mediated G1 arrest. Similarly, transfection of cells with Chk1 siRNA completely protected the cells from G1 arrest induced by piperine. Piperine treatment caused down-regulation of E2F1 and phosphorylation of retinoblastoma protein (Rb). Apoptosis induced by piperine was associated with down-regulation of XIAP, Bid (full length) and cleavage of Caspase-3 and PARP. Furthermore, our results showed that piperine treatment generated ROS in melanoma cells. Blocking ROS by tiron protected the cells from piperine mediated cell cycle arrest and apoptosis. These results suggest that piperine mediated ROS played a critical role in inducing DNA damage and activation of Chk1 leading to G1 cell cycle arrest and apoptosis.

## Introduction

Melanoma is a type of skin cancer and considered to be one of the major causes of death from skin diseases. The median survival time of the patient post diagnosis is 9 months with a 5 year survival probability of less than 5% [1]. Genetically melanoma is a very complex disease with the major involvement of Ras/Raf/MEK/ERK pathway. BRAF mutation is observed in majority of melanomas [2]. Several other genetic alterations observed in melanoma include mutation in NRAS, overexpression of Bcl-2, NF-_k_B and Akt-3 and loss of PTEN [3]. Previous studies have shown the role of Cyclin D-CDK4/6 in the phosphorylation of all the three pockets of Rb protein, leading to its inactivation [4]. Consequently, several E2F family members are present in an unbound and transcriptionally active form [5] [6]. Melanoma cells have a very low rate of spontaneous apoptosis and are notoriously resistant to the drugs *in vivo* and drug induced apoptosis *in vitro* [7]. Since there are several barriers in the efficient treatment of melanoma, novel approaches of targeting molecular pathways in melanoma are needed.

Piperine is an alkaloid extracted from black pepper (*P. nigrum*) and long pepper (*P. longum*). Previous studies have shown that piperine has anti-inflammatory, antiarthritic and anti-depressant effects [8] [9]. Piperine has also been known to inhibit CYP3A4 and P-glycoprotein due to which it has been used to enhance the bioavailability of other drugs [10]. When co-administered with curcumin, piperine increased the bioavailability of curcumin by 2000% [11]. In a clinical study, simultaneous administration of piperine with docetaxel enhanced the anti-tumor efficacy of docetaxel. Clinical trials are also being conducted to evaluate the effect of piperine in enhancing the bioavailability of resveratrol.

In the present study, we demonstrate the anti-proliferative effects of piperine in murine as well as in human melanoma cells. Our results demonstrate that piperine treatment caused ROS generation in melanoma cells and that ROS were involved in inducing G1 cell cycle arrest through the activation of Chk1, and apoptosis.

## Materials and Methods

### Chemicals

Piperine was obtained from LKT Laboratories (St. Paul, MN). Sulforhodamine B, RNase A, propidium iodide, ampicillin, NAC, actin antibody, and trichloroacetic acid were obtained from Sigma-Aldrich (St. Louis, MO). Electrophoresis reagents were from Bio-Rad Laboratories (Hercules, CA). Antibodies against phospho-Chk1 (Ser296), phospho-ATR, phospho-H_2_A.X (Ser139), phospho-Rb (Ser795), p21, E2F1, p53, XIAP, Bid (uncleaved), cleaved Caspase 3, cleaved PARP and human specific SignalSilence Chk1 siRNA kit were procured from Cell Signaling Technology (Danvers, MA). Antibody against Cyclin D1 was obtained from Abcam (Cambridge, MA) and antibody against DNA polymerase β was acquired from Neomarkers (Fremont, CA). Transfection reagent siPORT NeoFX was obtained from Ambion Inc (Austin TX). Trypsin, heat-inactivated fetal bovine serum (FBS) and penicillin/streptomycin antibiotic mixture were from Mediatech Inc. (Manassas, VA). Dulbecco’s Modified Eagle’s Medium (DMEM) and Eagle’s Minimum Essestial Medium (EMEM) were from the American Type Culture Collection (ATCC; Manassas, VA). Alexa Fluor 488 (anti-mouse), Alexa Fluor 594 (anti-rabbit) secondary antibodies and 2′,7–dichlorofluorescein diacetate (DCFDA) were acquired from Invitrogen (Carlsbad, CA). AZD7762 (Chk1 inhibitor) was purchased from Cayman Chemicals (An Arbor, MI).

### Cell Culture

SK MEL 28 and B16 F0 were a kind gift from Dr. Majid Moridani (Texas Tech University Health Sciences Center). A375 cells were provided by Dr. Tyler Wakenda (University of Rochester Medical Center, Rochester, NY). B16 F0 cells originated from C57BL/6J mice whereas SK MEL 28 and A375 cells were a malignant melanoma cell line obtained from a human male subject. Aspc-1 cells were purchased from ATCC (Manassas, VA). B16 F0 and AsPc-1 cells were cultured in DMEM medium supplemented with 10% FBS. SK MEL 28 and A375 cells were maintained in EMEM medium supplemented with 10% FBS. All the culture medium contained 1% penicillin-streptomycin-neomycin antibiotic mixture. The cell lines were maintained in a humidified incubator with 5% CO_2_/95% air. A 100 mM stock solution of piperine in Dimethyl Sulfoxide (DMSO) was prepared freshly before the experiment.

### Cell Survival Assay

About 5000 cells in 0.1 ml medium were seeded per well in a 96 well plate. After 24 hours of incubation, cells were treated with different concentrations of piperine and plates were incubated for 24, 48 and 72 hours. Cells were fixed using 10% tricholoroacetic acid (Sigma Aldrich Ltd.) and incubated for 1 hour at 4°C. Subsequently, cells were stained with 0.5% Sulforhodamine B solution and the absorbance were measured at 570 nm using a plate reader (BioTek Instruments, Winooski, VT) as described by us previously [12], [13].

### Cell Cycle Analysis Assay

Approximately 0.3×10^6^ cells were seeded in a 6 well plate. After 24 hours, cells were treated with different concentrations of piperine. After 48 hours, cells were collected and fixed with ice cold ethanol (70%) for 12 hours at 4°C. Cells were stained with propidium iodide and analysed using Flow Cytometry (Accuri C6) as described by us previously [14]. Approximately 2×10^4^ cells were analysed for each sample. Cell debris and clumps were excluded from the analysis in all samples.

### Annexin V-fluorescein Isothiocyanate (FITC) Apoptosis Assay

The apoptosis assay was performed using a kit (BD Biosciences, San Jose, CA, USA). Approximately, 3×10^6^ cells were seeded in a 6– well plate. After 24 hours, cells were treated with different concentrations of piperine for 48 hours. Following the treatment, the cells were processed according to the manufacturer’s instructions and analyzed using Flow Cytometry (Accuri C6). Cell debris and clumps were excluded from the analysis in all the samples.

### Western Blotting

B16 F0 and SK MEL 28 cells were treated with varying concentrations of piperine for the indicated time periods. Cells were washed twice with ice-cold phosphate-buffered saline and lysed as described by us previously (13). Protein content was determined using Bradford reagent and lysate containing 20 to 80 µg of protein was subjected to SDS gel electrophoresis followed by immunoblotting as described previously [12].

### Chk1 Inhibitor Treatment

In a separate experiment, SK MEL 28 cells were treated with 300 nM and 600 nM of AZD 7762 or 10 mM tiron for 1 hour at 37°C followed by treatment with 150 µM piperine for 48 hours. Subsequently, cells were processed for flow cytometric analysis or western blotting.

### Transfection of Cells with Chk1 siRNA

About 2×10^5^ SK MEL 28 cells were seeded in a 6-well plate and transfected with siRNA using siPORT as the transfection reagent. The reaction mixture was prepared in Opti-MEM serum-free media in which 100 nM of Chk1 siRNA was mixed with 8 µL of transfection reagent. This mixture was incubated for 30 mins after which it was added to the cells. The cells were incubated in the mixture for 5 hours and then replenished with normal growth media for 24 hours. Subsequently, cells were exposed to 150 µM piperine for 48 hours and processed for flow cytometry.

### Immunofluorescence

Immunofluorescence staining was performed according to the method described by us previously [15]. SK MEL 28 cells were plated in a 24-well plate on a cover slip at a density of 0.5×10^5^. They were allowed to attach overnight and further treated with 150 µM of piperine for 48 hours. The cells were then fixed with 4% paraformaldehyde and blocked with 1% goat serum and 0.25% Tween 20 in PBS for 1 hour. Cells were permeabilized using 0.05% Triton X in PBS followed by incubation with p.Chk1 and β-actin overnight at 4°C with constant shaking. Subsequently, the cells were incubated with Alexa Fluor 488 (anti-mouse) and Alexa Fluor 594 (anti-rabbit) secondary antibodies at room temperature with gentle shaking. Finally, the nucleus was stained with DRAQ 5 (Axora LLC, San Diego, CA, USA). The coverslips were then mounted on the slides and the images were evaluated under the microscope (Olympus Inc.).

### Determination of ROS Generation

Approximately 1×10^6^ cells were plated per well in a 6-well plate and allowed to attach overnight. Cells were then treated with varying concentrations of piperine for a pre-determined time period and then incubated with 10 µM DCFDA for another 30 mins. Cells were collected, washed with ice-cold phosphate-buffered saline (pH 7.4) and analysed using Flow Cytometer (Accuri C6).

### Tiron and NAC Treatment

In a separate experiment, SK MEL 28 cells were treated with 10 mM tiron or NAC for 1 hour at 37°C followed by treatment with 150 µM piperine for 48 hours. Subsequently, cells were processed for flow cytometric analysis, western blotting or sulphorhodamine B assay.

### Statistical Analysis

All statistical calculations were performed using Prism 5.0 (GraphPad Software Inc., San Diego, CA). Results were expressed as means ± S.D. of at least three independent experiments, each conducted in triplicate. Data were analyzed by Student’s *t* test or one-way analysis of variance followed by Bonferroni’s post hoc analysis for multiple comparisons. Differences were considered statistically significant at *p*<0.05.

## Results

### Piperine Suppresses the Survival of Melanoma Cells

**Figure 1:**
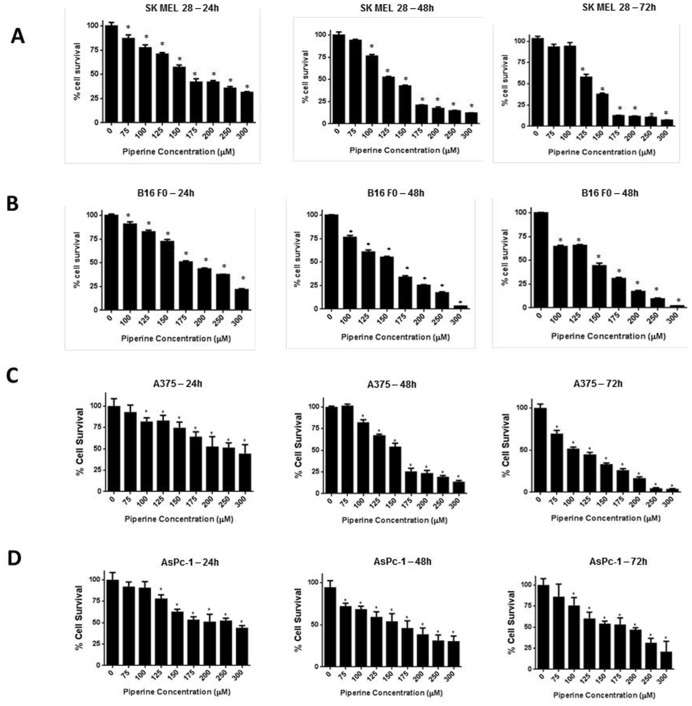
Piperine suppresses the survival of melanoma cells. Effect of various concentrations of piperine at different time periods in (A) SK MEL 28, (B) B16 F0, (C) A375 and (D) Aspc-1 cells was determined by Sulforhodamine B cell survival assay. Values are the means ± S.D. of three independent experiments with eight replicates; *p<0.05 when compared with control.

Firstly, we evaluated the effect of piperine on the growth of melanoma cells. For this purpose we used B16 F0, SK MEL 28 and A375 cells. Treatment with varying concentrations of piperine resulted in a significant growth suppression of all the cell lines (Fig. 1). The IC_50_ of piperine in SK MEL 28 was 221 µM, 172 µM and 136 µM at 24, 48 and 72 h of treatment whereas the IC_50_ of piperine in B16 F0 cells was found to be 200 µM, 155 µM and 137 µM at 24, 48 and 72 h of treatment respectively (Fig. 1A–B). Moreover, IC_50_ of piperine in A375 cells was 225 µM, 160 µM and 100 µM at 24, 48 and 72 h respectively (Fig. 1C). Also, our results showed that higher concentrations of piperine were able to suppress the growth of B16 F0 almost completely at 48 and 72 hours of treatment as compared to 90% in SK MEL 28 or A375 cells. Since melanoma cells are usually very resistant, we wanted to see whether other cell lines were more sensitive to piperine treatment or not. Hence, we also looked at the effect of piperine in AsPc-1 cells, a pancreatic cancer cell line. Our results showed that the IC_50_ of piperine in AsPc-1 cells was 250 µM, 195 µM and 180 µM at 24, 48 and 72 h (Fig. 1D). These results suggest that piperine suppress the growth of all the cancer cells in a concentration and time-dependent manner.

### Piperine Induces G1 Phase Arrest in Melanoma Cells

**Figure 2:**
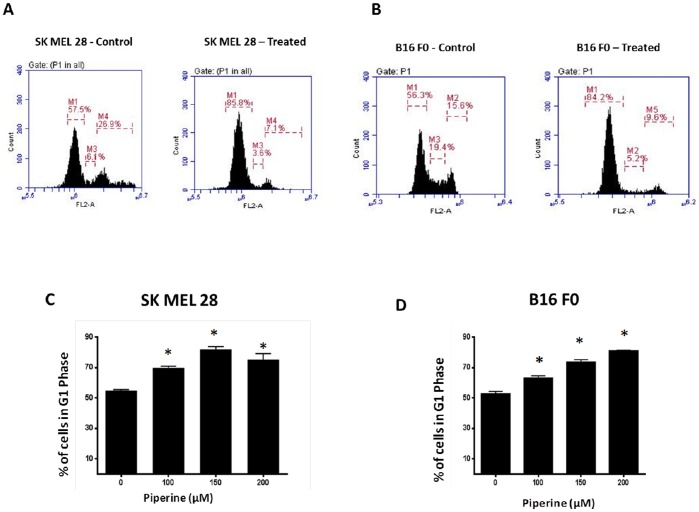
Piperine induces G1 phase cell cycle arrest in melanoma cells. (A) and (B) are representative cell cycle profiles of control and 150 µM piperine treated SK MEL 28 and B16 F0 cells for 48 h. FL2-A represents the intensity of propidium iodide, and the *y*-axis represents the cell counts. (C) And (D) represents concentration-dependent effects of piperine on number of cells in G1 phase in both SK MEL 28 and B16 F0 respectively. Values are means ± S.D. of three independent experiments, each conducted in triplicate. *p<0.05 when compared with control.

To identify the mechanism behind the cell growth inhibition, we determined the effect of piperine on cell cycle progression (Fig. 2). Cells were treated with various concentrations of piperine and analysed using flow cytometry. Our results showed that 150 µM piperine caused significant accumulation of SK MEL 28 and B16 F0 cells in G1 phase (Fig. 2A–B). There was a concentration dependent increase of cells in G1 phase with a concomitant decrease of the cells in S and G2/M phase (Fig. 2C–D). About 85% of B16 F0 cells were arrested in G1 phase. Similarly, SK MEL 28 cells when treated with 200 µM piperine for 48 hours resulted in 76% cell population in G1 phase. These results indicate that piperine treatment induces G1 phase arrest in melanoma cells.

### Piperine Causes DNA Damage in Melanoma Cells

**Figure 3:**
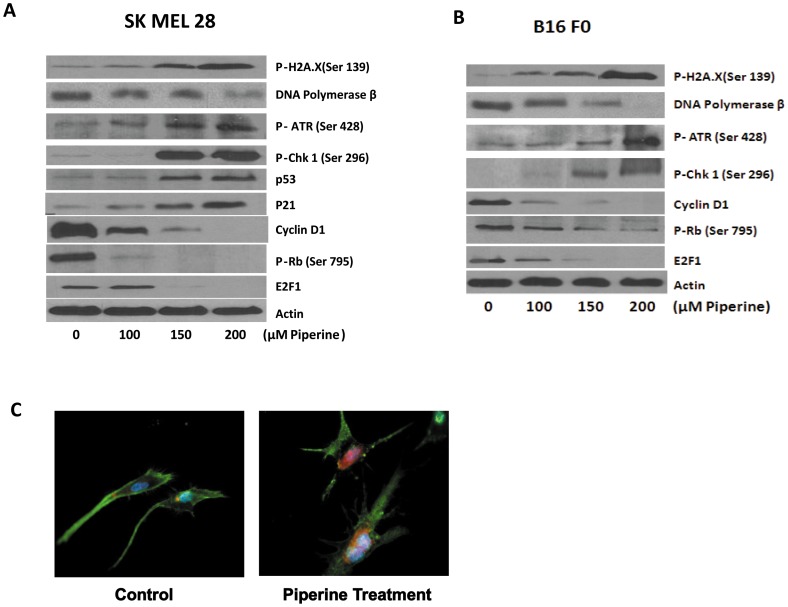
Piperine causes DNA damage and modulates G1 cell cycle regulatory proteins. SK MEL 28 (A) and B16 F0 (B) cells were treated with different concentrations of piperine for 48 h. Cells were lysed and total lysate was prepared as described under *Materials and Methods* and analyzed by western blotting. Representative immunoblots show the effect of piperine on the phosphorylation of H2A.X (Ser139), ATR (Ser428), Chk1 (Ser296) and p-Rb (Ser795), and the protein levels of DNA Polymerase β, p53, p21, Cyclin D1 and E2F1. Each blot was stripped and reprobed with anti-actin antibody to ensure equal protein loading. (C)Representative immunofluorescence images of p. Chk1 (Ser 296) in control and 150 µM piperine treated SK MEL 28 cells. Alexafluor 594 (Red) represents p.Chk1, Alexafluor 488(green) represents β-actin and DAPI (blue) represents nucleus. Each experiment was performed at least three times independently and the results were comparable.

To elucidate the molecular mechanism behind the arrest of melanoma cells in G1 phase by piperine, we subjected control and treated cells to western blotting. Previous reports from our lab have shown DNA damage to be a major inducer of cell cycle arrest [14], [16]. Our current results showed that piperine treatment significantly increased the phosphorylation of H2A.X at Ser 139, which is a marker of DNA damage (Fig. 3). The increase in phosphorylation of H2A.X was observed in a concentration dependent manner in both the cell lines. Moreover, we observed that piperine treatment drastically reduced the expression of DNA polymerase β, an enzyme which plays a very important role in the repair of DNA strand breaks (Fig. 3A–B). These results suggest that piperine causes DNA damage and prevents the repair of the damage.

### Piperine Modulates G1 Cell Cycle Regulatory Protein

Usually, in response to DNA damage, ATM/ATR and checkpoint kinases are activated. [16]. To delineate the molecular mechanism of piperine mediated G1 arrest, we determined its effect on the key DNA damage response proteins. Our results showed significant increase in the phosphorylation of ATR at Ser 428 in the cells treated with piperine (Fig. 3A and B). No change was observed in the phosphorylation of ATM (data not shown). There was a substantial increase in the phosphorylation of Chk1 at Ser 296 but not Chk2 (Fig. 3A–B). In addition, there was a marked decrease in the expression of cyclin D1 by piperine treatment (Fig. 3A–B). On the other hand, there was also a significant increase in the expression of p53 (Fig. 3A), which could be related to DNA damage and activation of ATR. An increase in the expression of p21^Cip1^, a Cyclin Dependent Kinase Inhibitor (CDKI) was observed in SK MEL 28 cells by piperine treatment (Fig. 3A). P21 is known to negatively regulate G1 transition. Furthermore, we looked at the modulation of the proteins in the dynamic complex of retinoblastoma (Rb) and E2F proteins, which are known to play an important role in G1 transition. Exposure of melanoma cells to piperine significantly reduced the phosphorylation of Rb protein at Ser795 (Fig. 3A and B). There was also a substantial decrease in the protein levels of transcription factor E2F1 (Fig. 3A–B). We further determined the phosphorylation of Chk1 upon piperine treatment by immunofluorescence. For this purpose, SK MEL 28 cells were treated with 150 µM piperine for 48 hours and analysed by immunofluorescence staining (Figure 3C). The red staining represents p.Chk1, green staining β-actin and the blue staining for nucleus. Significant staining of p.Chk1 was observed in the nucleus of piperine treated cells as compared to control (Fig. 3C). All these results show the involvement of ATR/Chk1/p53/p21 in piperine mediated G1 cell cycle arrest.

### Piperine Induces Apoptosis in Melanoma Cells

**Figure 4:**
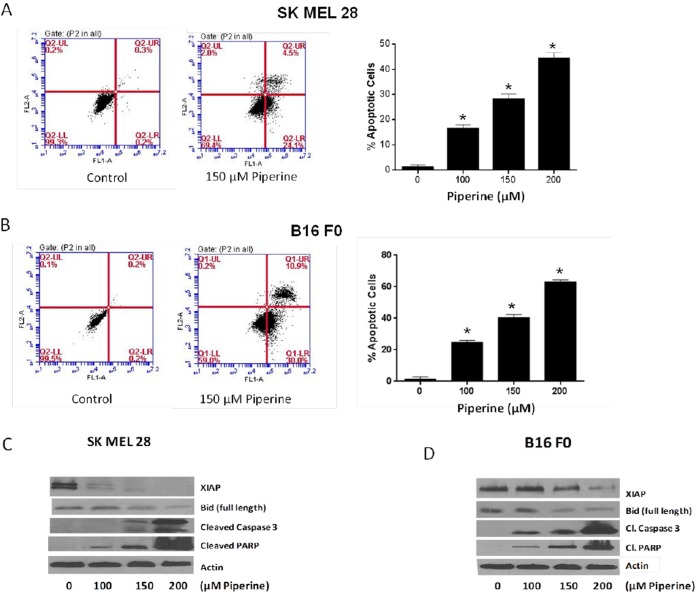
Piperine induces apoptosis in melanoma cells. SK MEL-28 and B16 F0 cells were treated with different concentrations of piperine for 48 h. Cells were stained with Annexin V and PI and analysed using flow cytometer. (A) and (B) shows representative apoptosis profile of SK MEL 28 and B16 F0 respectively. It also shows the concentration-dependent increase in the percent of apoptotic cells in both the cell lines. Figure (C) and (D) shows western blot analysis of SK MEL 28 and B16 F0 cell lysates upon piperine treatment respectively. Representative immunoblots show the effect of piperine on the protein levels of XIAP, Bid (full length), Cleaved Caspase 3 and Cleaved PARP. Each blot was stripped and reprobed with anti-actin antibody to ensure equal protein loading. Each experiment was performed at least three times independently and the results were comparable.

P53 is a known regulator of cell death through induction of apoptosis. Since we observed an increase in the expression of p53, we wanted to determine whether or not piperine induced apoptosis in melanoma cells. Hence, we performed an apoptosis assay using Annexin V-FITC. Our results revealed that piperine induced significant apoptosis in both the cell lines (Fig. 4A–B). Treatment of SK-MEL-28 cells with 150 µM and 200 µM resulted in about 30% and 45% apoptosis respectively (Fig. 4A). On the other hand, B16 F0 cells were more sensitive to piperine-induced apoptosis. Percentage of apoptotic cells in B16 F0 at 100 µM, 150 µM and 200 µM piperine concentrations were 25%, 40% and 60% respectively (Fig. 4B). To confirm these observations we looked at the expression of key proteins involved in apoptotic pathway upon piperine treatment by western blotting. The expression of XIAP, an inhibitor of apoptosis, and Bid (full length) were down-regulated by piperine treatment indicating mitochondrial death pathway (Fig. 4C–D). In B16 F0 cells, there was a decrease in the expression of Bcl-2 protein by piperine treatment whereas no such change was observed in SK MEL 28 cells (data not shown). On the other hand, in SK MEL 28 there was a substantial down regulation of Bcl-XL but no change was observed in B16 F0 (data not shown). In addition, piperine treatment caused significant cleavage of caspase-3 and PARP in both the cell lines indicating apoptosis (Fig. 4 C–D). These results clearly revealed piperine mediated induction of apoptosis in melanoma cells.

### Chk 1 Inhibitor Blocks Piperine Mediated G1 Arrest

**Figure 5:**
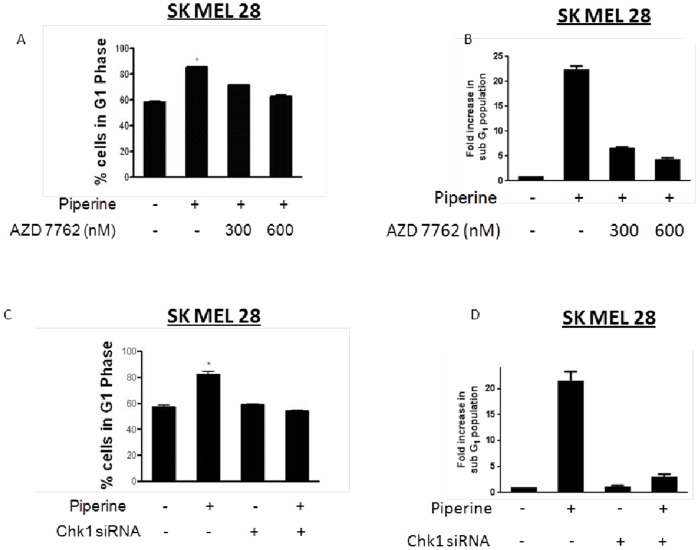
Blocking Chk1 activation suppress the effects of piperine. SK MEL 28 cells were (A) and (B) treated with AZD 7762 or (C) and (D) transfected with Chk1 siRNA prior to treatment with 150 µM piperine for 48 hours. Subsequently, cells were processed for flow cytometric analysis. Each experiment was performed at least three times independently and the results were comparable. Values are mean ± S.D of three independent experiments. *p<0.05 when compared with control, **p<0.05 when compared with piperine treatment.

Since we observed significant activation of Chk1 upon piperine treatment, we wanted to determine the role of Chk1 in cell cycle arrest induced by piperine. For this, we pre-treated SK MEL 28 cells with 300 nM and 600 nM AZD7762, a specific inhibitor of Chk1, and evaluated the effect of piperine in these cells. Our results show that AZD7762 blocked the activation of Chk1 by piperine and hence G1 cell cycle arrest in a concentration dependent manner (Figure 5A). AZD7762 (600 nM) was able to completely protect the cells from piperine mediated G1 cell cycle arrest. Moreover, upon treatment with Chk1 inhibitor along with piperine, cells that were arrested in G1 phase by piperine were redistributed between S and G2M phase giving a cell cycle profile similar to control cells. We also evaluated sub-G1 cells by flow cytometery by piperine treatment. As compared to control, piperine treatment increased sub-G1 population by 22 folds. However, sub-G1 cell population was reduced to 7 fold and 4 fold when the cells were treated with 300 nM and 600 nM AZD7762 respectively prior to treatment with piperine. These results suggest that inhibition of Chk-1 activation blocked piperine mediated apoptosis in melanoma cells (Fig. 5B).

### Chk1 siRNA Abrogates Piperine Induced G1 Arrest

To confirm the role of Chk1in piperine mediated G1 cell cycle arrest and apoptosis, we transiently silenced Chk1 in SK MEL 28 cells using Chk1 specific siRNA. It is important to note that Chk1 silencing completely blocked piperine mediated G1 cell cycle arrest in SK MEL 28 cells (Figure 5C). Furthermore, as compared to 22 fold in control, piperine was able to induce only 3 fold increase in sub-G1 cell population in Chk-1 silenced cells (Figure 5D). These results not only confirmed the critical role of Chk1 in piperine mediated G1 arrest but also showed a clear link between piperine mediated cell cycle arrest and apoptosis in melanoma cells.

### Piperine Generates ROS in Melanoma Cells

**Figure 6:**
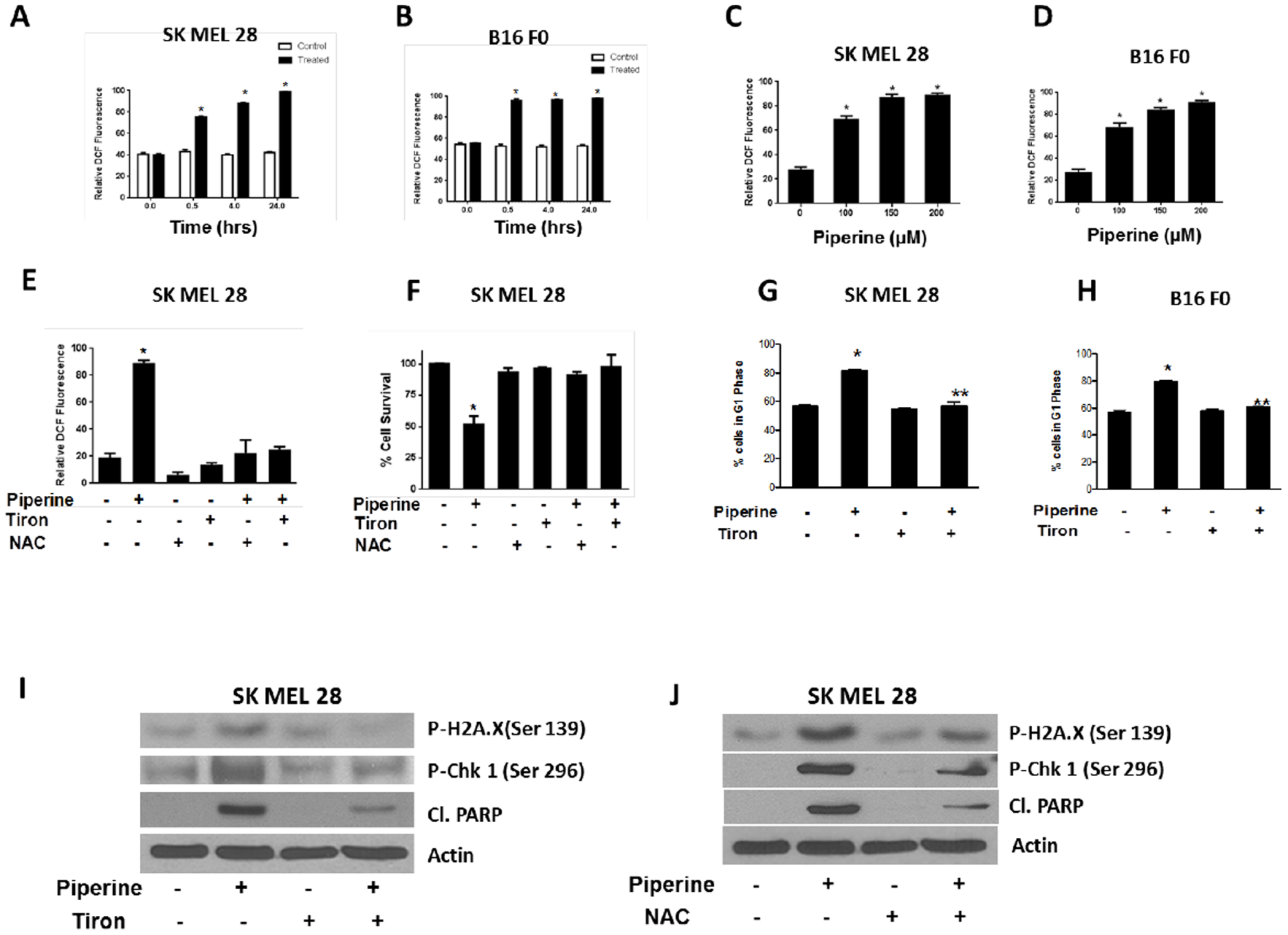
Piperine generates ROS in melanoma cells. (A) Represents time dependent generation of ROS in SK MEL 28 cells and (B) represents ROS in B16 F0 cells in response to 150 µM piperine treatment and subsequently analysed using flow cytometer. (C) SK MEL 28 and (D) B16 F0 cells were treated piperine following which the cells were analyzed for ROS using flow cytometer. (E) SK MEL 28 cells were pre-treated with either 10 mM tiron or NAC for 1 h and then treated with 150 µM piperine for 48 h. The cells were processed for ROS analysis by flow cytometry. (F) SK MEL 28 cells were pre-treated with either 10 mM tiron or NAC for 1 h followed by 150 µM piperine for 48 h after which the cell survival was evaluated by sulphorhodamine B assay. (G) SK MEL 28 and (H) B16 F0 cells were pre-treated with 10 mM tiron for 1 h followed by 150 µM piperine for 48 h. The cells were then processed for cell cycle analysis by flow cytometry. In another experiment, SK MEL 28 cells were pre-treated with (I) tiron or (J) NAC as described above and analyzed by western blotting for p.H2A.X, p.Chk1 and cleavage of PARP. Each experiment was performed at least three times independently, and the results were comparable. The values are means ± S.D. *p<0.05 when compared with control. **p<0.05 when compared to piperine treatment.

Next, we sought to determine the mechanism behind DNA damage and the activation of Chk1. Previous studies have shown the involvement of ROS in inducing DNA damage and cell cycle arrest [14], [17]. Therefore, ROS generation was determined using flow cytometer by measuring the fluorescence of DCF, which is formed due to the oxidation of DCFDA by endogenous peroxides. Early and persistent generation of ROS was observed by piperine treatment in both the cell lines. The level of ROS increased steadily in a time-dependent manner in both the cell lines (Fig. 6A–B). We also observed a concentration dependent induction of ROS upon piperine treatment. On a relative scale, the percentage of cells with DCF fluorescence in SK MEL 28 was 69, 87 and 90% and that in B16 F0 was 68, 84 and 91% when treated with 100, 150 and 200 µM piperine respectively (Figure 6C–D). In both the cell lines, percentage of cells with DCF fluorescence in control was around 27% (Figure 6C–D).

### Tiron and NAC Blocks DNA Damage, G1 Arrest and Apoptosis in Melanoma Cells

To confirm the involvement of ROS in piperine mediated G1 arrest, B16 F0 and SK MEL 28 cells were pretreated with antioxidants tiron or NAC prior to piperine treatment. As a proof of principle, we wanted to check whether tiron and NAC could block ROS induction upon piperine treatment. As expected, both tiron and NAC completely suppressed piperine induced ROS in SK MEL 28 cells (Figure 6E). The percentage of cells with DCF fluorescence was 20%, which increased to 90% with piperine treatment (Figure 6E). However, when the cells were treated with piperine in presence of tiron, the percentage of DCF positive cells went down to 25% and that in presence of NAC went down to 22% (Figure 6E). Next we evaluated the effect of both the antioxidants on the growth inhibitory effects of piperine. We observed that growth inhibitory effects of piperine were completely abrogated when SK MEL 28 cells were pre-treated with tiron and NAC (Figure 6F). There was a 50% growth inhibition of SK MEL 28 cells by piperine treatment. However, piperine failed to inhibit the growth of cells treated with tiron or NAC (Figure 6F). We further looked at the effect of antioxidant on piperine-induced cell cycle arrest. Our results demonstrated that tiron pre-treatment completely protected both SK MEL 28 and B16 F0 cells from piperine mediated G1 arrest (Figure 6G–H). Finally, both tiron and NAC treatment also blocked the activation of Chk1and H2A.X hence DNA damage (Figure 6I–J). There was also a decrease in the piperine-mediated cleavage of PARP in presence of tiron and NAC indicating abrogation of apoptosis by antioxidants (Figure 6I–J). In summary, these results suggest that ROS generated by piperine plays a very crucial role in inducing DNA damage, cell cycle arrest and apoptosis in melanoma cells.

## Discussion

Our results show that piperine suppressed the growth of SK MEL 28, B16 F0 and A375 cells in a time dependent as well as concentration-dependent manner. The growth suppression of these cells was due to G1 phase cell cycle arrest. Our results further showed that G1 arrest by piperine was linked with DNA damage and activation of Chk1eventually leading to apoptosis in melanoma cells. Furthermore, piperine treatment caused ROS generation and blocking ROS by antioxidant blocked the deleterious effects of piperine. To the best of our knowledge, this is the first study that establishes the growth inhibitory effect of piperine in melanoma cells through G1 phase cell cycle arrest.

There are several cell cycle checkpoints for the maintenance of normal cell cycle progression and to ensure the protection of dividing cells from of DNA damage. In response to DNA damage, cells are arrested in G1phase to prevent the defective cells progressing to S phase [18]. This provides time to the cells to repair the damage and proceed further to the next phase or enter into apoptosis if the damage is not repaired [19]. Our results demonstrated an increase in the phosphorylation of H2A.X at Ser139 by piperine treatment indicating DNA damage in these cells. Our results are in agreement with previous reports that have shown G1 arrest as a result of DNA damage and phosphorylation of H2A.X [20]. DNA polymerase β is a critical enzyme responsible for the repair of DNA strand breaks. Our results showed a significant decrease in the expression of DNA polymerase β in the cells exposed to piperine. DNA damage along with reduced ability to repair the damage could be the mechanism by which piperine caused G1 cell cycle arrest and apoptosis in melanoma cells.

ATM/ATR is activated in response to DNA damage [21]–[23]. These proteins upon activation get recruited at the site of damage and phosphorylate checkpoint kinases such as Chk1 and Chk2 [24]. There are two pathways, which regulate G1 phase cell cycle transition. The first pathway consists of the Chk1 phosphorylation by activated ATR, which in turn inhibits Cdc25A, causing its proteosomal degradation. Cdc25A is a phosphatase which dephophorylates the inhibitory phosphate groups on CDK4 or CDK/6. The second pathway is the activation of p53, which in turn activates p21 [18]. P21 is a universal cyclin dependent kinase inhibitor that inhibits the cyclin D-CDK4/6 complex that phosphorylate key proteins required for the progression of the cells to S phase [25]. Complex formation of CDK with cyclin is very essential for its kinase activity. Mutations in Chk1 have been frequently observed in many types of cancer causing genetic instability. The alteration in DNA damage checkpoint has been one of the reasons for resistance of tumors to chemotherapeutic drugs [26]. Usually, activation of Chk1 by ATM is responsible for G2/M cell cycle arrest by phosphorylation of Cdc52C at Ser 216. However, there are several reports which suggest the involvement of Chk1 in G1 phase cell cycle [27]. Enormous efforts have been made to understand the role of checkpoints in carcinogenesis. In response to DNA damage, Chk1 has been established as a transducer of ATM/ATR. Irregular function of Chk1 has been identified as one of the hallmarks of neoplastic transformation. Radiation therapy and chemodrugs have been shown to activate Chk1 leading to the arrest of cells [12], [28].

Our results demonstrate a significant increase in the phosphorylation of ATR at Ser 428 and Chk1 at Ser296, respectively suggesting DNA damage as the cause of initiation of cell cycle arrest. Blocking Chk1 activation by AZD 7762 (Chk1 inhibitor) or Chk1 siRNA protected the cells from piperine mediated cell cycle arrest. Immunofluorescence studies showed extensive activation of Chk1 at Ser296 and its nuclear localization in the cells treated with piperine. These results suggest that the activation of Chk1 and its nuclear localization is essential for piperine-mediated cell cycle arrest.

One of the main events in the progression of the cells from G1 to S phase is the activation of E2F-DP complex regulated by Cyclin-Cdk. Under normal condition, hypo-phosphorylated pRB binds to E2F causing its inactivation [29]. Cyclin D combines with CDK4/6 and hyper-phophorylates pRB, which leads to its dissociation from the E2F complex hence, permitting the transcription of key S phase promoting genes. Our results show a marked down regulation of Cyclin D1 indicating the decreased activity of CyclinD1-CDK4/6 complex. Further, reduced phophorylation of Rb at Ser795 by piperine treatment further suggests the inhibition of Rb hyper-phosphorylation. Moreover, decrease in the expression of E2F1 by piperine indicates repression of E2F complex. Interestingly, studies have shown that G1 arrest, loss of pRb and E2F also lead to cell senescence. However, piperine treatment did not cause any cell senescence as no β-galactosidase (β-gal) staining or change in the expression of p16^INK4A^ was observed in our model (data not shown). β-gal and p16^INK4A^ are considered to be the hallmarks of cell senescence. In summary, all these results clearly indicate that piperine modulates G1 phase proteins resulting in the arrest of melanoma cells.

The cell cycle arrest gives sufficient time to the cells to repair damaged DNA. In case of irreparable damage, cells proceed to apoptosis. Our results show a significant cleavage of caspase-3 and PARP upon piperine treatment. Furthermore, down-regulation of XIAP and Bid (full length) also suggest induction of apoptosis in the cells exposed to piperine. Reduction of cells in sub-G1 phase by AZD7762 or Chk-1 siRNA in combination with piperine in our studies shows a link between piperine-mediated cell cycle arrest and apoptosis.

Earlier studies have shown the involvement of ROS in causing DNA damage [14], [16]. Consistent with these observations, ROS generation was observed within 30 minutes of piperine treatment in both the cell lines and the levels persisted upto 24 hours. ROS generation by piperine was concentration dependent in melanoma cells. Interestingly, antioxidants tiron and NAC blocked the phosphorylation of histone H2A.X as well as Chk1 and consequently cell cycle arrest. NAC and Tiron also decreased the cleavage of PARP hence blocked piperine-mediated reduced survival of melanoma cells.

Taken together, our study showed a direct link between the ROS generation, Chk-1 activation through DNA damage leading to G1 cell cycle arrest and apoptosis. Nevertheless, further studies are required to identify other proteins playing role in the overall growth inhibitory effects of piperine in melanoma cells.
